# Individual differences in reading social intentions from motor deviants

**DOI:** 10.3389/fpsyg.2015.01175

**Published:** 2015-08-17

**Authors:** Daniel Lewkowicz, Francois Quesque, Yann Coello, Yvonne N. Delevoye-Turrell

**Affiliations:** SCALab, UMR CNRS 9193, Department of Psychology, Université de Lille, Villeneuve-d’Ascq, France

**Keywords:** perception, action, social cognition, intention, observation, kinematics

## Abstract

As social animals, it is crucial to understand others’ intention. But is it possible to detect social intention in two actions that have the exact same motor goal? In the present study, we presented participants with video clips of an individual reaching for and grasping an object to either use it (personal trial) or to give his partner the opportunity to use it (social trial). In Experiment 1, the ability of naïve participants to classify correctly social trials through simple observation of short video clips was tested. In addition, detection levels were analyzed as a function of individual scores in psychological questionnaires of motor imagery, visual imagery, and social cognition. Results revealed that the between-participant heterogeneity in the ability to distinguish social from personal actions was predicted by the social skill abilities. A second experiment was then conducted to assess what predictive mechanism could contribute to the detection of social intention. Video clips were sliced and normalized to control for either the reaction times (RTs) or/and the movement times (MTs) of the grasping action. Tested in a second group of participants, results showed that the detection of social intention relies on the variation of both RT and MT that are implicitly perceived in the grasping action. The ability to use implicitly these motor deviants for action-outcome understanding would be the key to intuitive social interaction.

## Introduction

Understanding what a conspecific is doing represents a crucial ability for our everyday social interactions. However, perceiving an action and understanding the reason that drives this behavior may arise from different processes (Spaulding, 2015). As highly social species, it is crucial for us to perceive others’ mental states and to predict what they plan to do in order to adapt and coordinate our own behavior to the surrounding context (Hamilton, 2009; Sebanz and Knoblich, 2009). As such, our ability to understand the goal of others’ actions relies on a variety of sources (Frith and Frith, 2006). For example, declarative knowledge (Fehr and Fischbacher, 2004) and indirect interaction (Singer et al., 2004) are indices that are used when judging the reason of others’ behavior. Contextual cues, such as environmental and physical constraints of an action also help to detect the aim of observed actions (Brass et al., 2007; Stapel et al., 2012). However, experimental evidences now support the hypothesis that humans have the ability to predict the action-outcome goals on the basis of the observation of its early kinematics only (Orliaguet et al., 1996; Knoblich and Flach, 2001; Sebanz and Shiffrar, 2009). Indeed, it has been shown that observers are sensitive to early differences in visual kinematics and can use them to discriminate between movements performed with different object-oriented motor intentions (Méary et al., 2005; Manera et al., 2011; Sartori et al., 2011). However, it is the case that most gestures are socially oriented: I can reach for a cup and place it on a table in order to use it myself, but often I will reach for an object to give it to my partner. The question that will be considered here is then: Can my partner detect in a predictive manner whether the cup that I am grasping for is for her or not, simply by observing my hand moving?

After considering the literature that discusses how intentions may shape movement kinematics, we will state the differences that are rarely made between motor and social intentions in experimental settings. More specifically, we will reveal the individual differences in the ability to detect social intention when simply observing the motor deviants contained within 3D movement kinematics.

Kinematic studies in humans have shown that different motor intentions can shape the spatio-temporal characteristics of a reach-to-grasp movement depending on the goal of the executed sequence (Marteniuk et al., 1987; Armbrüster and Spijkers, 2006; Ansuini et al., 2008; Naish et al., 2013). For example, people tend to produce slower motor actions when grasping an object with the intention to place it accurately rather than with the intention to throw it (Marteniuk et al., 1987; Louis-Dam et al., 1999). In addition, Jacob and Jeannerod (2005) distinguished two types of intentions. The motor intention refers to the mental state that causes the execution of voluntary action (e.g., to put a glass on a table). However, the same motor intention could involve a conspecific (e.g., put the glass on a table for your child) or not (e.g., put the glass on the table to drink from it). This later level of description is referred to as the social intention that is, the intention to affect a conspecific’s behavior. According to these same authors, only the motor intention influences the execution of an action, since the same spatial constraints could serve different social intentions. This is known in the literature as the Dr. Jekyll and Mr. Hyde paradox (Jacob and Jeannerod, 2005). Interestingly, recent studies have shade doubt on these affirmations by showing that specific changes in the kinematics of the arm and hand movements can be revealed when investigating the effects of the social context on the execution of motor sequences (Ferri et al., 2011a; Gianelli et al., 2011; Innocenti et al., 2012; Scorolli et al., 2014). But more specifically, it has been suggested that when endorsing a social intention, humans tend to amplify the spatio-temporal parameters of their movements. When planned with a social intention in mind, a subject’s hand tends to move with higher hand paths (Becchio et al., 2008; Quesque et al., 2013; Quesque and Coello, 2014), slower velocities (Becchio et al., 2008; Lewkowicz et al., 2013) and longer movement durations (Ferri et al., 2011b; Quesque et al., 2013; Quesque and Coello, 2014). From these variations in execution, it could then be possible for an observer to distinguish different social goals driving similar motor actions.

In the present contribution, we defined the kinematic *deviances* due to social intentions as the systematic difference between the kinematic features [e.g., movement time (MT), peak velocity, peak height] of two executed movements that have the same motor constraints (e.g., start and stop position, object shape, target shape, object initial, and final position) but executed for different social intents. The use of common kinematic features of movements is an important step for researchers to quantify accurately the deviances due to social intentions (Ansuini et al., 2014). Nonetheless, we underline that our definition of the kinematic *deviance* is not restricted to a specific parameter. Rather, we hypothesize that it is a mechanism that affects multiple components of the movement and its preparation. Thus, the expression of kinematic *deviance* in terms of specific kinematic features could vary depending of the type of action, the target object position and shape, and various other motor constraints. In other words, when changing the motor constraints of an action, one would also change its social *deviance*. Hence, to characterize the kinematic *deviance* due to social intention one needs to disentangle the multiple kinematic features to determine the potential candidates. By controlling precisely the external constraints of executed movements in real-time (Lewkowicz and Delevoye-Turrell, 2015), it is possible to verify that the significant deviances of kinematic features are not due to specific motor constraints but rather to internal determinants (see also Ansuini et al., 2015), which would give scientific basis for a better understanding of the Dr. Jekyll and Mr. Hyde paradox (Jacob and Jeannerod, 2005). Whereas it has already been shown that the early deviants of kinematic features could be directly exploited to help detect the underlying intention of an observed action (Sartori et al., 2011; Lewkowicz et al., 2013), it is still unclear whether the sensitivity to kinematic deviances is in relationship with the motor expertise or the social skills of the external observer.

A number of recent studies have shown that motor training directly influences action observation (Hecht et al., 2001; Casile and Giese, 2006). Especially in the case of very skilled observers, for example, in sports (Abernethy and Zawi, 2007; Abernethy et al., 2008; Aglioti et al., 2008), and artistic activity (Calvo-Merino et al., 2005, 2006), experts systematically outmatch novices in recognizing and predicting the outcome of observed action but also in detecting deceptive intentions (Jackson et al., 2006; Cañal-Bruland and Schmidt, 2009; Sebanz and Shiffrar, 2009). These results are in line with the hypothesis that common codes for perception and action (Prinz, 1997; Hommel et al., 2001) can be used to simulate observed actions and thus, gain a better prediction and understanding of motor outcome (Blakemore and Decety, 2001; Jeannerod, 2001; Wolpert et al., 2003; Grush, 2004; Wilson and Knoblich, 2005; Uithol et al., 2011). In addition, within the framework of the mirror neuron system (Cattaneo and Rizzolatti, 2009), it has been claimed that the same mechanisms would be involved during the imagery of a motor act directed to an object and the actual execution of that same motor act (e.g., Jeannerod and Decety, 1995; Ehrsson et al., 2003; Decety and Grèzes, 2006). The ability to detect social deviants should then be correlated to motor expertise and simulation abilities.

The processing of others’ movements is also very important for communication and adaptive social behavior. Individuals who exhibit deficits in visual biological motion processing are also compromised on daily-life social perception (see Pavlova, 2012, for a review). When one interacts with another person, it is assumed implicitly that common thoughts are shared. Thus, in social contexts, we unconsciously spend time predicting the behavior of others on the basis of what we would do ourselves in the same situation. One may up to a certain extent try to place our own self within the other person’s mind, beliefs and desires. This complex cognitive function is referred to as having a “theory of mind” (Premack and Woodruff, 1978), taking an intentional stance (Dennett, 1987), or mentalizing (Frith, 1989). Mentalizing has been studied using a wide range of tasks including reading stories (Fletcher et al., 1995; Saxe and Kanwisher, 2003), looking at cartoons (Brunet et al., 2000; Gallagher et al., 2000), and watching simple animations (Castelli et al., 2000). It has recently been proposed that during action observations the neural basis of the “theory of mind” is more recruited when the observer is explicitly asked to interpret the scene in terms of high-level goals than it is when focusing on lower-level intentions (Van Overwalle and Baetens, 2009). In such a case, recognizing social deviants may be associated to the same mechanisms, which participate in the recognition process of body and face for social cognition.

In the current study, our goal was to test whether by maintaining the motor intention identical an observer is still able to dissociate between social and personal intentions in movements performed toward an object. After recording trials of actors performing social and personal reach to grasp actions and verifying that the kinematics were indeed dissociable, we conducted two experiments of action observation in which the participants’ task was to categorize trials as a function of their social scope. In *Experiment 1*, we were specifically interested in the individual differences that may be observed in the ability to read social intentions. In order to gain an insight in the psychological factors that may be involved in the capacity of participants to understand the social action-outcome, we used questionnaires to capture both social cognition and motor imagery abilities. In *Experiment 2*, we probed the *nature* of the kinematic deviances of observed movements, which contributed to the categorizing of social and personal intentions. For this, we used post-recording treatments in order to control precisely for the amount of temporal information available within the movie clips. Through the alterations of specific properties of 3D motion kinematics, we were able to test the effects of a progressive normalization of deviances on the participants’ ability to categorize the action as being personal or social.

## Experiment 1: Inter Individual Differences to Recognize Social Patterns

In this first study, we tested whether the ability to recognize social intention through the decoding of social deviants was related to motor imagery and/or social cognition abilities.

### Materials and Methods

#### Participants

Twenty-five healthy young adults (seven males; mean age: 24.7; SD: 3.0) participated in the experiment. All had normal or corrected-to-normal vision and had no prior knowledge of the experimental goals. They gave informed consent before participating in the experimental session that lasted approximately 30 min. The protocol received approval from the ethics committee for Human Sciences of the University of Lille 3.

#### Apparatus and Stimuli

***Stimuli***

To create the experimental material, we filmed two naïve adults seated at a table, facing each other, and participating in a short cooperative game. The game consisted in displacing a little wooden dowel (width 2 cm; height 4 cm) between the thumb and the index finger to different locations. Their sequential actions were time-locked to a series of broadcasted sounds. The first move of the game was always performed by the same member of the dyad (named here, the “actor”) and consisted in displacing the dowel from an initial location to a central target. After this preparatory action, a subsequent main action was to be performed either by the actor (*personal* condition) or by the partner (*social* condition). Two blocks of 15 trials were performed: In one block, the actor performed all the preparatory and the main actions, the partner being just an observer. In the other block, the actor performed the preparatory actions and the main actions were always performed by the partner. Meanwhile, the actor’s movements were recorded using a video camera (Logitech webcam model c270) to record the scene. In addition, four Oqus infrared cameras (Qualisys system) were used to record the upper-body kinematics. Five infrared reflective markers were placed on the index (base and tip), the thumb (tip), the wrist (scaphoïd and pisiform) of the actor; one marker was placed at the top of the object. The calibration of the cameras provided the means to reach a standard deviation smaller than 0.2 mm, at a 200 Hz sampling rate.

**FIGURE 1:**
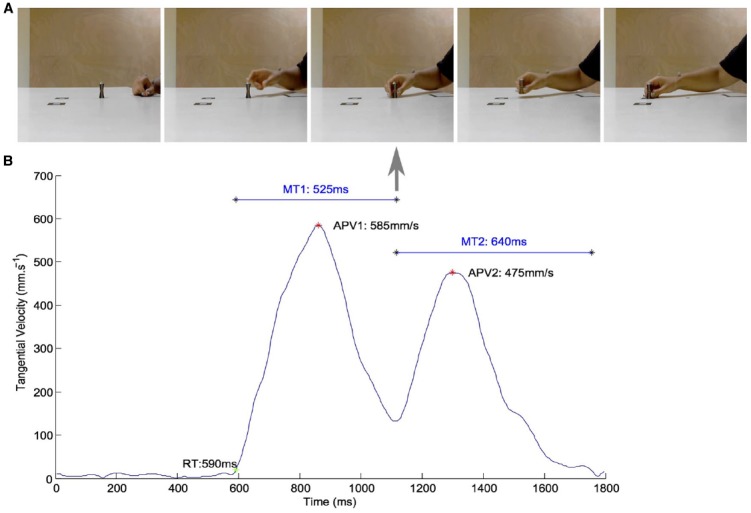
**(A)** A typical example of the video stimuli that was used both in Experiments 1 and 2 to test the role of motor deviants for the categorization of social and personal object-centered actions. One can note the neutral context that was used with the placement of 3D reflexive markers that provided us the means to verify the kinematic deviants between social and personal movements during the validation phase of the video database. **(B)** Velocity curves of the corresponding trial illustrating the double bell shaped profiles that are observed in the present reach to grasp task. Reaction times (RT in ms) and movement times of the first element of the sequence (MT of reach in ms) may have been used by the observers to dissociate social from personal actions.

**FIGURE 2:**
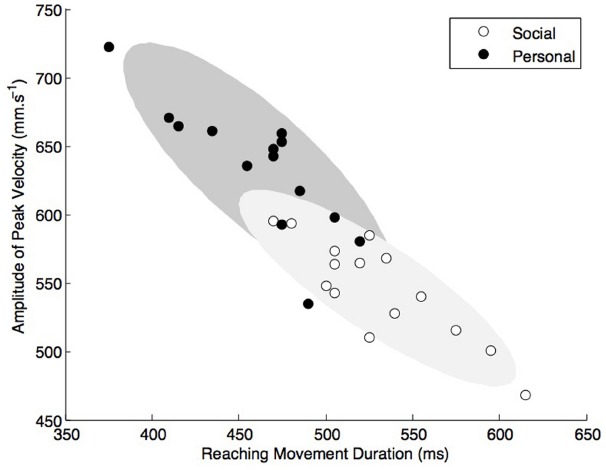
**Kinematic deviances are presented with the amplitude of peak velocity plotted as a function of movement time.** Scatterplots reveal none negligible proportions of the plots that are discriminative and thus, may be used to dissociate social from personal intention.

A particular attention was taken to suppress all contextual information from the video clips (see Figure 1A). Only the arm of the actor and the target object were framed within the video clips of the 30 preparatory actions. The video clips that were used as stimuli consisted in a sequential action of two motor elements (1) reach to grasp and (2) move to place. The video clips were cut exactly one frame after the actor finished placing the object. Movies were compressed with FFdshow codec (MJPEG) at 30 frames per second with a screen resolution of 640 × 480 pixels. 3D kinematics were analyzed with RTMocap toolbox (Lewkowicz and Delevoye-Turrell, 2015). Positional data points were filtered using a dual fourth-order Butterworth low-pass filter (fc = 15 Hz; forward and backward) and tangential 3D instantaneous velocities were calculated. A threshold of 20 mm·s^–1^ was used to determine the onset of movement (reaction time, RT). All velocity trajectories were bell shaped and consisted in two “bells,” the first corresponding to the reach to grasp element, the second being the move to place element of the preparatory action. The amplitude of peak velocity of the first element (APV_1_) was extracted using the local maxima (first 0-crossing of acceleration). The end of the first element was determined as the time of occurrence of the local minima (second 0-crossing of acceleration) between the first and the second element-peaks (see *vertical arrow* in Figure 1). The duration of the first element (MT_1_) was calculated as the time interval between the onset and the end of the first element. The amplitude of the peak height of trajectories (APH_1_) was defined as the maximum z coordinate of the wrist measured in the grasping element and the lift to place element. APV_2_, MT_2_, and APH_2_ are the corresponding kinematic parameters described above but extracted from the second move to place element of the motor sequence. Table 1 presents the characteristics of the movement parameters that were measured, e.g., RT, MT, peak wrist velocity, and height of hand trajectory. Figure 2 presents the scatterplot of amplitude of peak velocity against MT in order to confirm none negligible proportions of the plots that are discriminative between social and personal trials. Using comparison to the median values, pre-analysis confirmed the possibility to dissociate personal from social trials on the basis of RT, MT and height of grasping phase (APH).

**TABLE 1:** **Mean kinematic parameters of the preparatory action for both the personal and the social trials**.

	**RT**	**APV1**	**APV2**	**MT1**	**MT2**	**APH1**	**APH2**
	**	*	*	***	*	**	*
Personal intention	616	582	525	440	508	58	63
Social intention	702	547	457	478	545	60	65
Median values	665	572	487	457	533	59	64
Frequency of personal trials > median	4/15	10/15	5/15	3/15	5/15	4/15	5/15
Frequency of social trials > median	11/15	5/15	10/15	12/15	10/15	11/15	10/15

For each parameter, the median values for the totality of the trials are reported and the frequency of trials superior to this value is specified in each condition. RT, reaction time; APV, amplitude of peak velocity; MT, movement time; APH, amplitude of peak hand height, for the first (1) reaching element or the second (2) grasping element. The asterisks revealed the parameters for which significant differences were found between the two distributions in the personal and the social conditions using the median test (*p < 0.05; **p < 0.01; ***p < 0.001).

***Individual evaluations of social and imagery sensitivity***

The *Reading the Mind in the Eyes* Test, which will be referred to as the RME-test in the following sections (Baron-Cohen et al., 1997, 2001) was designed to measure each individuals’ sensitivity to social cues and in particular the participants’ ability to understand others’ complex mental states. This test has shown a high potential to distinguish an individual’s tendency to attend to others’ intentions in joint cognitive tasks (Ruys and Aarts, 2010). In the RME-test, participants were required to categorize eye-regions of 36 facial expressions by selecting a mental state label that matched the perceived expression, selecting one out of the four terms proposed. In the present experiment, participants completed a French version of this test (Prevost et al., 2014) and were encouraged to select the appropriate term as fast as possible. Overall, the more people attend to the intentions of others, the higher are their scores on the RME-test. We also administered a French version (Loison et al., 2013) of the Movement Imagery Questionnaire—Revised Second version (MIQ-RS, Gregg et al., 2010) of the Movement Imagery Questionnaire—Revised (MIQ-R, Hall and Martin, 1997). This questionnaire is a reliable measure of motor imagery that distinguishes kinesthetic motor imagery from visual motor imagery. Participants were required to perform and imagine daily life actions that were similar in the two subscales, involving both upper and lower limbs.

#### Procedure

Participants were seated at a table in a silent experimental box, facing the experimenter. They took part in a short cooperative game to get familiarized with the paradigm. These pre-test trials consisted in similar manipulative movements than that performed by the actor in the stimuli video. Participants performed 15 trials for which they were required to pick and place a wooden dowel at the center of the table for their own purpose and 15 trials for which the wooden dowel was picked and placed for the experimenter. After this familiarization phase, participants were instructed to watch and categorize previously recorded videos clips from the same two conditions. Participants had to categorize a total of 30 videos (15 social and 15 personal). The instructions before categorization were given orally as follow (“*Is the actor placing the dowel for a personal use?” OR “Is the actor placing the dowel to give it to his partner?*”).

The videos stimuli in the categorization task were displayed on a gray background on a laptop computer using the *PsychToolbox* for Matlab (Natick, MA, USA). Before each trial, a white fixation cross-appeared on the gray screen during a variable interval of 500–1000 ms. After each video presentation, as soon as the clip ended, a blank screen was shown during which participants were prompt to give their decision. They were instructed to categorize each movie clip as fast and as accurately as possible. The response keys were marked with tape placed directly on the azerty computer keyboard (“a” for social and “p” for personal). The response keys were counterbalanced across participants. No feedback was given during the experiment. Finally, the participants were required to complete the French version of the RME-test and the MIQ-R. The order of presentation of the two tests was also counterbalanced across participants. After the entire completion of the experiment, participants were asked to comment on the general degree of confidence that they had in their answers in the categorization task. Finally, participants obtained a short debriefing period and were thanked for their participation.

#### Analysis

Response times were calculated as the time interval between the presentation of the last frame of the video and the participant’s key press. For the analyses of the amount of correct responses, it is to note that in our experiment the error in judging one kind of stimulus (e.g., social) was redundant with the correct judgment of the other kind of stimulus (e.g., personal). Consequently, the results were expressed in total percentage of correct responses (Bond and DePaulo, 2006). Scores for each category were compared to the reference constant, i.e., the random answer value of 0.50, with a single sample *t*-test. To test whether the classifications rates would entail any substantial individual differences in the perception of social intention, we performed correlation analyses. We then checked whether the percentage of correct responses was correlated with the social cognition measure and with the motor and visual imagery measures, separately. Final score in the French version of the *RME-test* was computed on 34 items, excluding the items 13 and 23 from analysis as recommended (Prevost et al., 2014). Concerning the imagery measures, the two scores (kinesthetic; visual) were calculated on a 7 points scale. All analyses were conducted two-tailed and the alpha level of significance was set to 0.05.

### Results

#### Categorization Performance and Response Time

The results revealed that on average participants were able to categorize the underlying intention above chance level (*M* = 65.7%, SD = 15.8 vs. 50%), *t*(24) = 4.980, *p* < 0.001. There were no significant differences in the percentage of correct categorization for the personal intention (*M* = 68%, SD = 19.7) and the social intention (*M* = 63.4%, SD = 19.8), *t*(24) = 0.95, *p* = 0.35. Moreover, the results revealed no significant effects of the stimulus type on mean response times. Participants categorized the video clips presenting a personal intention as quickly (*M* = 600 ms, SD = 0.39) as the video clips presenting a social intention (*M* = 570 ms, SD = 0.32), *t*(24) = 0.58, *p* = 0.58.

#### Correlation With Individual Traits

**FIGURE 3:**
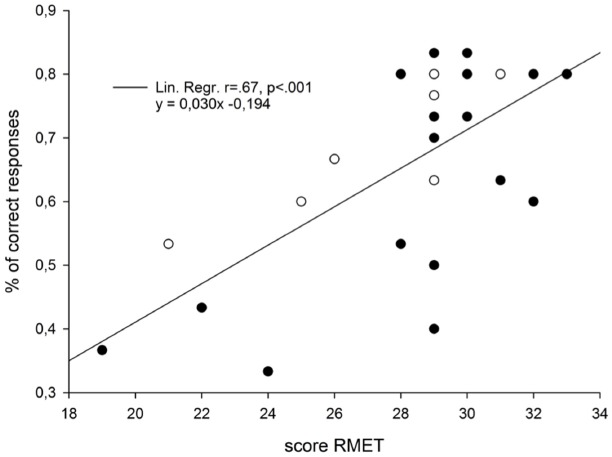
**Graphical illustration of the correlation parameters that were obtained in Experiment 1 between the individual scores of the ***Read the Mind in the Eyes*** Test (RME-test), and the percentage of correct answers given in the categorization task.** Black dots and white dots represent female and male participants, respectively.

On average, participants obtained a score of *M* = 5.8, SD = 1.2 in visual imagery and *M* = 4.8, SD = 1.3 in kinesthetic imagery as assessed by the Movement Imagery Questionnaire. The results revealed an absence of correlation with the percentage of correct categorization for both the visual imagery score (*R* = 0.125, *p* = 0.551) and the kinesthetic imagery score (*R* = 0.194, *p* = 0.354). The results of the RME-test revealed a mean score of 28.24, SD = 3.5. Our results showed that the RME-test scores were positively correlated with the percentage of correct categorization (*R* = 0.677, *p* < 0.001), indicating that a higher score in the RME is associated to a higher performance in the categorization task (see Figure 3). Concerning the degree of relationship between the questionnaires, the RME-test scores were related neither to the kinesthetic imagery scores (*R* = 0.006, *p* = 0.975) nor to the visual imagery scores (*R* = 0.278, *p* = 0.178). Finally, the scores on the two dimensions of the Movement Imagery Questionnaire were not correlated (*R* = 0.132, *p* = 0.527).

### Discussion

The aim of this experiment was to test for the individual differences that may be observed in the ability to read social intentions. Firstly, confronted to short video clips of “pick and place” moves, participants were able to categorize the intention (“social” vs. “personal”) of the actor above chance level. Given the effort made to produce stimuli presenting an absence of contextual information, this result confirms the idea that not only motor intention (Méary et al., 2005; Manera et al., 2011; Sartori et al., 2011; Lewkowicz et al., 2013) but also social intention can be inferred from the kinematics of a movement, as suggested by Ansuini et al. (2015). Secondly, it is to note that not all participants were equally talented in performing the task. Particularly, the ability of participants to discriminate between social and personal intentions was highly linked to the scores obtained in the social cognition test but was not related to the scores obtained in the motor imagery questionnaires. Such dissociation corroborates recent findings showing that sensitivity to use subtle cues in biological motion is linked to social but not to motor imagery measures (Miller and Saygin, 2013). More specifically as reported here, the authors showed that form cues correlated more with the social than with the imagery measures suggesting that even if social cognition and motor imagery predict sensitivity to biological motion, these skills tap into different aspects of perception. In our case, the results comfort the idea that social abilities help detect modulations of trajectories even in very simple and fast motor actions such as a reach to grasp task performed at natural speed.

Experiment 1, gave us the opportunity to assess participants’ ability to perceive social intentions from motor actions. However, it did not give us insights on the actual perceptual cues used by participants to solve the decision task. Consequently, in Experiment 2, we focused on the question of “how” participants could perceive social intentions from motor actions. For this purpose, we used post-recording modifications of videos clips in order to determine which crucial aspects of the kinematic deviants were relevant for participants in making their categorization decision. Finally, during the debriefing sessions of Experiment 1 the vast majority of participants reported that they felt as if they responded randomly in the categorization task, reporting a very low degree of confidence in their responses. However, due to the absence of quantitative measures of the meta-cognitive judgments from the participants, it was not possible to draw straight conclusions. Experiment 2 gave the opportunity to investigate this point more rigorously by obtaining systematic auto-evaluation of metacognitive knowledge through the use of analogical-scales.

## Experiment 2: Content Information to Recognize Social Patterns

This study was conducted to assess whether participants could distinguish between social and personal movements even after the specific properties of the 3D motor kinematics were flattened out.

### Materials and Methods

#### Participants

Twenty-three healthy young adults (six males; mean age: 25.8; SD: 5.0) participated in the second experiment. All had normal or corrected-to-normal vision and had no prior knowledge of the experimental goals. These participants did not take part in Experiment 1 and gave informed consent before participating in the experimental session that lasted approximately 20 min. All participants completed in a previous session the French version of the RME-test (Prevost et al., 2014) and only those who had a minimal score of 27 (corresponding to the French median score) were selected to take part in the following experiment. The protocol received approval from the ethics committee for Human Sciences of the University of Lille 3.

#### Apparatus and Stimuli

In this experiment, two-step actions were recorded from a different actor but following the same design as in Experiment 1 in order to generate new stimuli videos. Table 2 presents the characteristics of actions parameters in the personal and social condition. As expected, significant differences were obtained in the 3D motion kinematics between personal and social trials for many motor parameters and especially those that will be manipulated, i.e., RT and MT of the first element of the motor sequence (MT1).

**TABLE 2:** **Mean kinematic parameters of the preparatory action for both the personal and the social trials**.

	**RT**	**APV1**	**APV2**	**MT1**	**MT2**	**APH1**	**APH2**
	*		*	**			**
Personal intention	395	590	529	417	501	58	58
Social intention	438	618	487	451	475	63	65
Median values	408	599	509	438	485	61	63
Frequency of personal trials > median	5/15	9/15	11/15	4/15	9/15	6/15	4/15
Frequency of social trials > median	10/15	6/15	5/15	11/15	7/15	9/15	11/15

The asterisks revealed the parameters for which significant differences were found between the two distributions in the personal and the social conditions using the median test (*p < 0.05; **p < 0.01).

In order to control for the amount of temporal and kinematic information available to participants, we used post-recording modification of the videos. This manipulation led to creation of three types of stimuli. Indeed, depending on the condition, the stimuli that were displayed could be the original video clips (*RT + MT_1_ deviant*), video clips normalized according to RTs (*MT_1_ deviant*) or video clips normalized according to the end of the grasping action (*No deviant*).

The modification of each video clip was achieved on-line as follows. First, the mean of the parameters that needed to be homogenized was calculated across all trials (social and personal). Second, the video clips were displayed at an overall refreshment rate so that the display time of this parameter corresponded to the mean pre-determined value. For example, in the MT_1_ deviant condition, the parameter that needed to be homogenized was the RT. Thus, using the kinematic data, a deviance ratio was calculated for the section of the video clip corresponding to the overall rate at which the RT section of the video should be presented in order to match the mean pre-determined value. We then interpolated the video frames (30 hz) with the true refreshment rate of the screen (60 hz) and replaced each video frame accordingly to the deviance ratio scaled to this final refreshment rate. In other words, the modifications brought to the duration of each video clip was spread out through the successive frames rather than being performed through an abrupt modification a given section of the video (e.g., by removing a frame). This manipulation gave us the opportunity to maintain the majority of the biological content of each movement.

Except for the modifications brought to the videos, the experimental design was identical to the one used in Experiment 1. In addition, analogical scales (10-cm long lines coding for “chance level” to the far left and “high confidence” to the far right) were included at the end of each trial in order to gain information about the metacognitive knowledge that participants’ possessed on their self-evaluation performances.

#### Procedure

Participants were seated at a table in a silent experimental box and had to perform the categorization task with the same instructions as in Experiment 1. They categorized the three sets of videos in three distinct sessions that were completed in a random order (counter-balanced across participants). After each session, they were asked to auto-evaluate the trust they had in their present classification rate on analogical scales.

#### Analysis

Mean percentages of correct responses, mean response times and mean self-evaluation scores were calculated for each condition and submitted to a repeated-measure ANOVA with condition (*RT + MT_1_ deviant*, *MT_1_ deviant*, *No deviant*) as within factors. The *post hoc* Bonferroni test was used when needed. We also conducted sub-analyses for the percentages of correct responses: scores for each category were compared to the reference constant, i.e., the random answer value of 0.50, using a single sample *t*-test. All analyses were conducted two-tailed and the alpha level of significance was set to 0.05.

### Results

**FIGURE 4:**
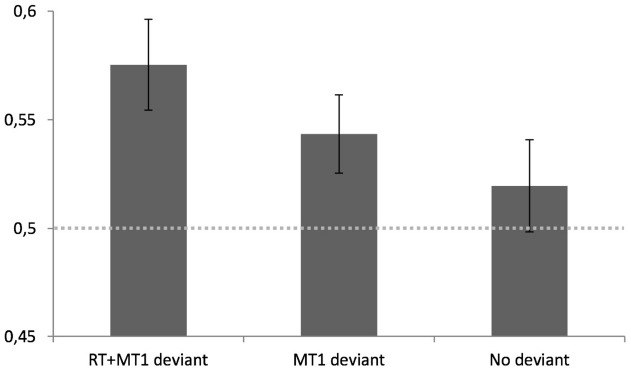
**Mean percentage of correct categorization for the three experimental conditions with standard errors (bars).** Note that when post-treatment of the videos were performed to normalize both reaction times (RT in ms) and the movement times of the first reach element of the sequence (MT1 in ms), participants were not able to categorize the social intention of the visual kinematics above chance level (illustrated here by the gray dotted horizontal line).

A repeated measures ANOVA revealed an effect of video type [*F*(1,22) = 3.02, *p* = 0.05] on the percentage of correct categorization. *Post hoc* contrast analysis revealed a significant higher rate of correct judgments in the natural condition (*M* = 57.5%, SD = 10) compared to the RT + MT_1_ deviant condition (*M* = 51.9%, SD = 10; *t* = 2.32, *p* < 0.05). Furthermore, the performances in the MT_1_ deviant condition were located in the middle range (*M* = 54.3%, SD = 08) not differing statistically from the two other conditions (*t* = –0.22, *p* = 0.83), suggesting a progressive decrease across the three experimental conditions. Two-sided *t*-tests comparing performances against chance level (50%) in the categorization task revealed that participants were significantly above chance in two of the three conditions (see Figure 4). More specifically, participants were able to categorize the underlying intention above chance level when videos were presented in the RT + MT_1_ deviant condition [*t*(22) = 3.6, *p* < 0.01] and in the MT_1_ deviant condition [*t*(22) = 2.4, *p* < 0.05]. However, they were not able to respond above chance level when videos were presented in the No deviant condition [*t*(22) = 0.9, *p* = 0.37].

Concerning response times, we found no significant effects of video type [*F*(1,22) = 2.19, *p* = 0.15]. Furthermore, the participants’ responses on the analogical scales used to evaluate metacognitive knowledge about performance self-assessment did not differ between conditions [*F*(2,44) = 0,02, *p* = 0.98]. With an overall mean of 68%, these observations indicate that participants found the task feasible but did not explicitly judge that a certain type of video was harder to categorize than another.

### Discussion

The driving question in the second study was to replicate those findings presented in Experiment 1 and assess to what extent kinematic deviants may be used to discriminate social intention in actions that have an exact same motor goal. As in study 1, participants were thus presented with short video clips and were asked to categorize the social intention of the actor. However, these video clips contained different amounts of informative deviants as the videos could be totally informative (original videos as in Experiment 1), partially informative (videos were normalized to RTs) or none informative (videos were normalized to the end of the grasping action). Using video clips of a different naïve actor, we replicated here the results reported in Experiment 1: individuals are able to distinguish between social intention and personal intention through the simple observation of motor kinematics. The fact that the overall categorization performance in the second study was lower than that seen in the first study could be due to the present of fewer kinematic deviances in the stimuli material. It is the case that when comparing trials in the social and the personal conditions, the kinematic analyses revealed more differences in Experiment 1 than in Experiment 2. It is true that in daily social interactions, the actions of certain individuals are easier “to read” than others. This situation—that we all have experienced, is reflected here by the fact that the actor who participated in Experiment 2 had kinematic variances that were less marked than the one participating in Experiment 1. Thus, our findings suggest that the kinematic signature of social intention is difficult to detect within a unique individual. Nevertheless, even if the amount of kinematic information was less present in Experiment 2, we were still able to cancel out the participants’ ability to read social intention through the modification of the kinematic features. Hence, social intention—even if weak, is contained within the kinematic variances of body movement.

The second important result that confirmed our initial hypothesis of the importance of motor deviants for intention reading was that the percentage of correct identification was proportional to the amount of deviants contained within motor kinematics. The original clips were better categorized than those stimuli that were partially normalized, suggesting that the categorical decisions were based on a spatio-temporal integration of that information contained within the actor’s movements. By asking participants to use analogical scales to self-evaluate performance levels, we furthermore showed that performance levels are not dependent on an explicit conscious decision process. Indeed, even if the percentage of correction identification was significantly affected by the deterioration of the video content, the participants’ metacognitive judgment was not. Participants did not explicitly detect differences in the informative values of the video clips and furthermore, did not judge their performance in the categorization task as being better or worse as a function of the informative content of the videos. Overall, these findings reveal the implicit nature of motor deviants to facilitate social interaction and confirm previous results found in the social literature suggesting that contextual information modulates social behaviors outside of awareness (Knoblich and Sebanz, 2008).

## General Discussion

Previous behavioral studies have revealed that the context in which object-oriented actions take place and their relevance for human interactions can affect the way very simple actions are executed (Ferri et al., 2011a; Gianelli et al., 2011; Innocenti et al., 2012; Scorolli et al., 2014). In the present contribution, we were interested in assessing the effects of social context on the temporal and the spatial parameters of hand trajectory in the basic action of reaching for and grasping an object, either to move it for self directed purposes (personal intention) or for the use of the object by a partner (social intention). Our question was the following: Could a naïve observer of the scene detect that the object was going to be reached with a social intention? What in the behavioral dynamics could be used as social cues? This experimental situation is very similar to that observed in daily experiences for which many of our interactions with conspecifics are not conveyed through language. For instance, it has been shown that both structural and dynamic information of body movement through space and time are taken into account for the recognition of point light-display of moving humans (Troje et al., 2005), or for the recognition of another’s emotions when the facial expression is not visible (Atkinson et al., 2004; Meeren et al., 2005). Likewise, in the present contribution, we showed that it is possible for a naïve observer to understand social intention of individuals performing an object-oriented motor action.

Movies were taken from a situation in which a participant picked up and placed an object knowing in advance whether herself or a partner will perform the next action in the sequence. With this method, we created stimuli in which kinematic variants (RT, MT and trajectory height) were the only factor conveying social meaning. Even though the kinematic *variations* due to social intention were small (a few millimeters within a few tens of milliseconds), motor deviants were present in our trajectories in a very repetitive and distinctive way (see Figure 2) confirming other experimental results reported in social oriented tasks (Becchio et al., 2008; Quesque et al., 2013). Here, we confirm in two different sets of actors that human observers are able to exploit these very small kinematic deviances to discriminate the social intention above chance level.

In Experiment 1, we focused on the personal determinants, which could explain inter individual differences in the ability to read the social intention of an action. We thus hypothesized that intention reading would be associated to an individual’s competence to either infer complex mental states to others or to use motor imagery to predict motor outcome from movement kinematics. We only found a positive correlation with the social skill as it was previously reported with biological motion processing (Miller and Saygin, 2013). The existence of a close relation between social abilities and the perception of social intention is not surprising as such. Whereas healthy adults are able to perceive intentions (Runeson and Frykholm, 1983; Blakemore and Decety, 2001) and emotions from point-light displays (Dittrich et al., 1996; Pollick et al., 2001; Atkinson et al., 2004; Grezes et al., 2007), this ability seems to be clearly impaired in patients showing deficits in social interactions such as in autism (Blake et al., 2003; Freitag et al., 2008; Parron et al., 2008; Cook et al., 2009; Centelles et al., 2012) and schizophrenia (Kim et al., 2005, 2011). The question that remains is then why does the correct discrimination of social intention not correlate with the motor imagery ability of the observer? We found that increased ability in motor imagery does not in itself help participants to understand correctly the social intention of the movement. One possible interpretation is that the motor imagery questionnaire probes more heavily the explicit processing of motor activity (e.g., goals, conscious monitoring) rather than the implicit sensitivity to subtle kinematic variations.

In Experiment 2, we focused on the hypothesis according to which observers may be able to read the social intention through the exploitation of the kinematic *deviances* between two movements executed with the same motor intention but different social intention. With post-recording treatments, we impoverished the temporal aspects of visual kinematics contained within the video clips to cancel out the ability to read social intention, confirming the central role of these temporal deviants in predicting social outcome. It is now generally accepted that when we execute a movement, we predict the sensory consequences of that movement through generative or forward models (Wolpert et al., 1995, 2003; Wolpert and Miall, 1996). These predictions can then be used to refine motor control problems induced by delayed feedback and sensory noise, but can also play a role to determine the most likely outcome of an observed action (Kilner et al., 2007). It has recently been suggested that a similar system can be used to understand others mental states (Oztop et al., 2005) and more specifically intentions (Ansuini et al., 2015). The results presented here confirm this hypothesis by showing that without temporal deviants, individuals lose the ability to categorize social outcome. These findings indicate that predictive timing may also be the key to the ability of decoding social intention through the observation of motor kinematics. Interestingly, break points were also relevant: RT normalization (in MT_1_ deviant condition) was here shown to also decrease categorization accuracy. This is congruent with previous studies that have shown that individuals are able to infer the subjective confidence of another person simply through the observation of RTs (Patel et al., 2012). Hence, those cognitive states that are based on predictive temporal properties may be correlated to social skills. Future studies need now to generalize these ideas and confirm that social reading is dependent on the accumulation of prediction errors, i.e., not only on *the when* but also on the *how long* of an on-flowing action sequence. Here we suggest that this would be done through the multi-integration of temporal deviants within a bilateral interaction of top-down and bottom up processes (see also Hillebrandt et al., 2014, for a neuro-anatomical account of this perspective).

It is the case that studies have reported gender effects related to social reading (Alaerts et al., 2011; Sokolov et al., 2011). Our results could suffer from the fact that a greater number of female individuals participated in the study. However, the gender main effect was none significant with the male participants performing at similar levels than the female participants both in the RMET and in the categorization task (see Figure 3). Furthermore, the tendency for woman to do better than men in the RMET was significantly true in the first version of the test (Baron-Cohen et al., 1997) but this was only marginally the case in the second version of the test (Baron-Cohen et al., 2001), which is the one we used. Finally, recent studies assessing the gender question have shown that men even sometimes do better than woman, e.g., in tasks using point-light displays to recognize human locomotion (Krüger et al., 2013). Hence, our results indicate that individual characteristics are more valuable to predict within gender abilities than the general gender property itself. They are novel and confirm the usefulness of RMET for predicting individual performances in (1) the recognition of body language (Alaerts et al., 2011; Miller and Saygin, 2013) and (2) the ability to detect other’s intention through body movements (Ruys and Aarts, 2010), whether that person be a man or a woman. A second point to note is the importance in future studies to assess whether the results presented here can be generalized to more ecological tasks. Indeed, the method presented here using video clips could be further applied to create experimental situations at second-person perspective including, for instance, two participants performing a reach to grasp task in a real interactive situation (see illustrated examples on line through reference keys given in Lewkowicz and Delevoye-Turrell, 2015). Furthermore, demonstrating that similar patterns of results are obtained when not only two but multiple intentional possibilities are presented would provide more ecological validity for the social abilities reported in the present study (see Obhi, 2012).

In conclusion, the present study reveals that the ability to implicitly use motor deviants from observed object-directed actions represents the crucial factor for detecting social intention. Furthermore, this ability seems to depend on individual social cognition skills. Implicit judgments are often considered as intuitive. As such, intuition has been defined in the field of human robotics as our ability for direct knowledge, for immediate insight without explicit reasoning. Intuitive judgments are more or less accessible to individuals depending on a number of factors (e.g., physical salience, emotional and motivational states, Kahneman, 2003). In the present study, we suggest that an important aspect of intuitive interaction is the power to detect the contained information within the temporal aspects of body movements to prime the social expectancy of an observer.

### Conflict of Interest Statement

The authors declare that the research was conducted in the absence of any commercial or financial relationships that could be construed as a potential conflict of interest.
